# 3D Assessment of Mandibular Growth Based on Image Registration: A Feasibility Study in a Rabbit Model

**DOI:** 10.1155/2014/276128

**Published:** 2014-01-02

**Authors:** I. Kim, M. E. Oliveira, W. J. Duncan, I. Cioffi, M. Farella

**Affiliations:** ^1^Department of Oral Sciences, University of Otago, P.O. Box 647, Dunedin 9054, New Zealand; ^2^Robotic System Laboratory (LSRO), Swiss Federal Institute of Technology in Lausanne (EPFL), 1015 Lausanne, Switzerland; ^3^Department of Oral Sciences, University of Naples Federico II, 80131 Naples, Italy

## Abstract

*Background.* Our knowledge of mandibular growth mostly derives from cephalometric radiography, which has inherent limitations due to the two-dimensional (2D) nature of measurement. *Objective.* To assess 3D morphological changes occurring during growth in a rabbit mandible. *Methods.* Serial cone-beam computerised tomographic (CBCT) images were made of two New Zealand white rabbits, at baseline and eight weeks after surgical implantation of 1 mm diameter metallic spheres as fiducial markers. A third animal acted as an unoperated (no implant) control. CBCT images were segmented and registered in 3D (Implant Superimposition and Procrustes Method), and the remodelling pattern described used color maps. Registration accuracy was quantified by the maximal of the mean minimum distances and by the Hausdorff distance. *Results.* The mean error for image registration was 0.37 mm and never exceeded 1 mm. The implant-based superimposition showed most remodelling occurred at the mandibular ramus, with bone apposition posteriorly and vertical growth at the condyle. *Conclusion.* We propose a method to quantitatively describe bone remodelling in three dimensions, based on the use of bone implants as fiducial markers and CBCT as imaging modality. The method is feasible and represents a promising approach for experimental studies by comparing baseline growth patterns and testing the effects of growth-modification treatments.

## 1. Introduction

An understanding of normal and abnormal mandibular growth has important implications for the diagnosis and management of craniofacial dysmorphism and syndromes [1]. Mandibular growth is the result of a heterogeneous pattern of bone resorption and apposition [2]. For proper interpretation of the biological and clinical significance of bone remodelling, morphological changes of the mandible must be described in relation to structures that are not affected by the remodelling process. The most reliable method for assessing mandibular growth is based on the use of subperiosteal implants as fiducial markers [3]. Our current knowledge of the extent to which the mandible changes and rotates during growth mostly derives from classical longitudinal studies that are based on serial two-dimensional (2D) cephalometric radiographs superimposed on bone markers [4, 5]. This approach, however, has questionable accuracy, is prone to measurement error, and the information obtained is limited by the 2D nature of the image [6, 7]. The introduction of three-dimensional (3D) radiographic imaging with cone-beam computed tomography (CBCT) now allows quantitative and qualitative analysis of bone remodelling that could not be attempted with standard 2D radiographs, enabling researchers to revisit the original concepts of growth [8–10].

Similar to cephalometric tracings, 3D models constructed from CBCT scans can be superimposed by registering common stable landmarks or by best fit of stable or minimally variant anatomical regions. The resulting displacement fields can be used to assess morphological changes in the regions of interest. The validity of 3D superimposition, however, still relies on stability of the reference structures. Although, some anatomical structures suggested to be stable on 2D radiographs during growth [3, 11] may not be valid for 3D evaluations. The mandibular canal, for instance, shifts laterally during growth and therefore is unsuitable for superimposition [9]. This highlights the relevance of additional information gained from 3D imaging and suggests that landmarks originally considered stable for 2D cephalogram superimpositions may not be applicable to 3D data sets. Furthermore, unlike the rest of the craniofacial complex, the mandible moves independently from the upper head and it requires segmentation, registration, and analysis independently. Hence, a biologically valid registration of serial 3D images of the mandible during growth requires implanted bone fiducial markers. The lack of robust methods for assessing mandibular growth in 3D can account to explain why there are so many controversial issues and unanswered questions surrounding clinical treatment modalities for craniofacial growth disorders.

We designed a pilot study to test the feasibility of an animal model aiming to assess 3D morphological changes of the mandible during growth, by superimposition of serial CBCT images of the mandible, based on Björk's traditional radiographic implant method.

## 2. Materials & Methods

The protocol was reviewed and approved by the Animal Ethics Committee of the University of Otago, New Zealand (AEC number 85/10).

### 2.1. Animals

Three, twelve-week-old, male New Zealand white rabbits (*Oryctolagus cuniculus*) were used in the study. During the study period, the animals were kept in individual pens and had access to water and food *ad libitum.* Body weight was registered at study commencement and daily throughout the 65 days of study period. Two rabbits underwent surgery for implant placement. The third rabbit was used as an unoperated (no implant) control and received no surgery.

### 2.2. Surgical Procedure

**Figure 1:**
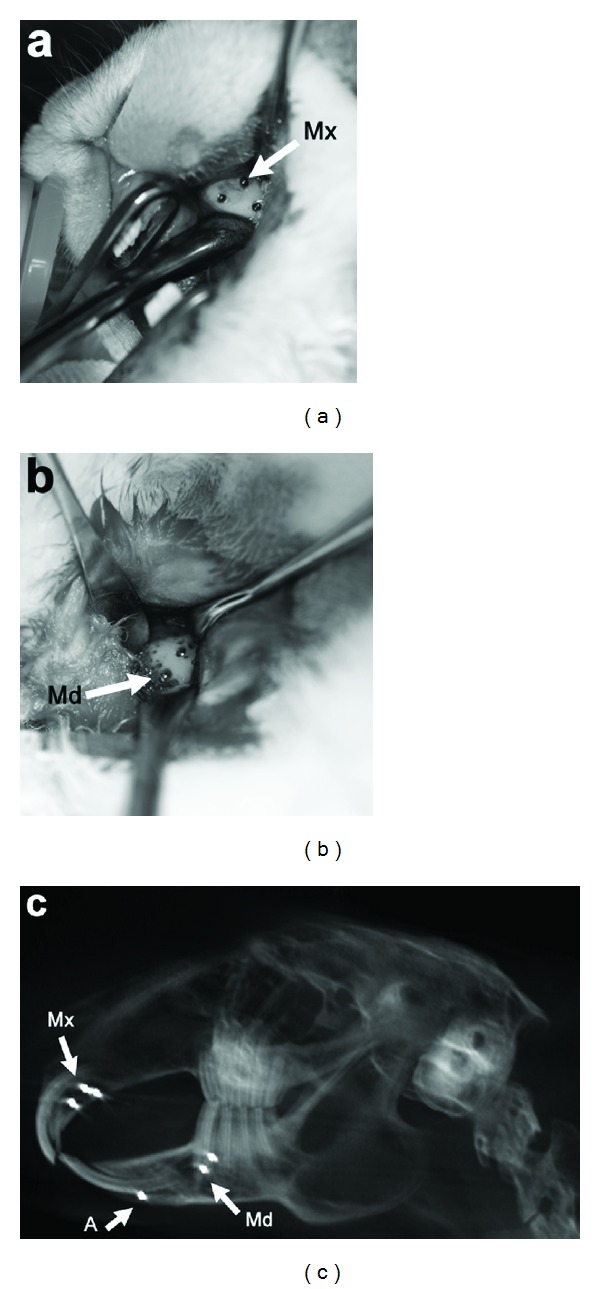
Surgical photographs and baseline radiograph showing placement of the metallic fiduciary markers. (a) Placement of 1 mm diameter tantalum spheres into the incisive bone of the maxilla (white arrow, Mx); (b) placement of spheres into the body of the mandible (white arrow, Md); and (c) baseline lateral cephalometric radiograph generated from cone-beam computerised tomographic volume, showing implants in the maxilla (Mx), body of the mandible (Md), and anterior mandible (A).

Metallic bone markers were used to aid with accurate superimposition of sequential radiographic images. Two rabbits had six or seven 1.0 mm diameter spherical tantalum implants (X-Medics, Denmark) inserted under general anaesthesia into the maxilla and mandible, to serve as radiographic landmarks. Anaesthesia was induced using subcutaneous Ketamine (25 mg/kg; Parnell Laboratories NZ Ltd.) and Domitor (0.5 mg/kg; Pfizer Animal Health) and maintained after intubation using halothane gas. Mepivacaine 2% local anaesthetic (Scandonest) with 1 : 100,000 adrenaline was administered prior to the surgical incisions to achieve local anaesthesia and haemostasis. The locations for implants were as follows: one in the mandibular-symphysis; two or three placed unilaterally into the lower border of the mandible, inferior to the molar teeth; and three placed anteroposteriorly along the incisive bone in the maxilla. Implants were placed under sterile conditions. In the mandibular symphysis region, an intraoral full thickness semilunar mucoperiosteal flap, 5 mm long, was raised in the depth of the buccal sulcus. This exposed the alveolar bone near the apex of the mandibular incisors (*Pars incisiva of Corpus mandibulae*). In the maxilla, the flap was located intraorally on the lateral surface of the incisive bone (*Corpus ossis incisivi*), lateral to the apex of the incisor tooth. A 1 cm linear incision was made and a full thickness mucoperiosteal flap elevated (Figure 1(a)). In the posterior mandible (*Corpus mandibulae*), a horizontal 1 cm long straight incision was made extraorally in the skin located at the anterior border of the masseter muscle, approximately 1 cm superior to the inferior border of the mandible. This incision was then dissected medially down to the bone, lateral to the apex of the first and second mandibular premolar teeth (Figure 1(b)).

In each of the three sites, an osteotomy was created in the bone using a 1 mm diameter drill bit at low speed with copious saline irrigation. The tantalum sphere was tapped into place, using a custom-made placement instrument. The final positions of the implants in the three surgical sites can be seen in Figure 1(c). A resorbable haemostatic gelatin sponge (Spongostan, Denmark) was applied to cover the exposed bone and implants, and the surgical field was closed in layers using 5-0 resorbable sutures (©Ethicon Inc., USA). Following the surgical procedures, anaesthesia was reversed using Antisedan (2.5 mg/kg; Pfizer Animal Health) and the animal was given subcutaneous carprofen anti-inflammatory analgesic (4 mg/kg; Norbrook) and enrofloxacin antibiotic (Baytril, 10 mg/kg; Bayer NZ Ltd.) twice daily for one week to prevent postoperative infection.

### 2.3. Three-Dimensional Imaging

**Figure 2:**
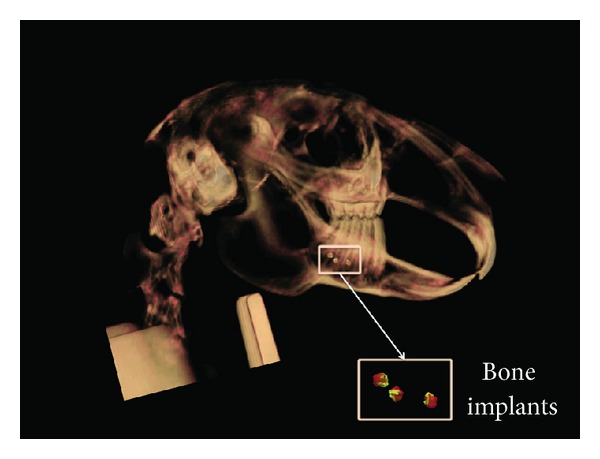
Three-dimensional rendering of CBCT imaging of a rabbit skull. The rectangular box indicates the position of bone implants into the mandible. The implants were used as an invariant feature for the registration of serial images of the mandible obtained during growth.

**Figure 3:**
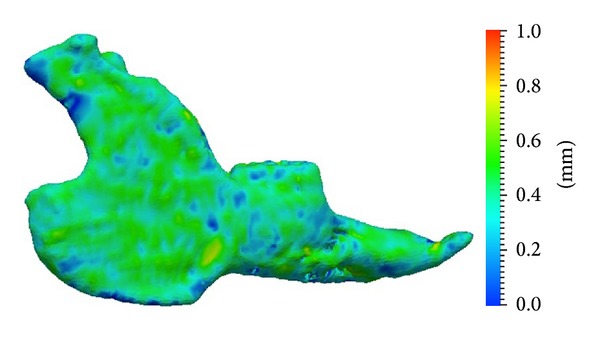
Scalar field representing minimum Euclidean distances between two surface meshes of the mandible extracted from one rabbit. Note the low errors resulting from image registration procedures.

At every imaging session, sedation was obtained by means of a subcutaneous injection of Ketamine/Dormitor (20/0.25 mg/kg) and reversed using Antisedan. A radiolucent vertical-cephalostat was designed to support and restrain the animal's body and head during exposure of images; this was fabricated by the Electromechanical Technology Workshop at Otago University. The animal was suspended vertically in a Galileos Comfort CBCT X-ray unit (Sirona; Bensheim/Germany), with the head supported by a radiolucent neck brace (Figure 2), such that only the head lay within the imaging volume encompassed by the horizontally rotating C-arm of the machine. Care was needed to avoid respiratory distress when the animal was suspended in this way. Full-head CBCT scans were taken at baseline and then at week 10 for each subject. The imaging protocol involved 6-second scans with a 15 cm^3^ field of view. The exposures were made at 85 kVp and 10 mAs with a 3D resolution isotropic voxel size of 0.3/0.15 mm. The Sidexis XG software package (version 2.52; Bensheim/Germany) and Galaxis 3D Viewer (version 1.7.2.1; Bensheim/Germany) were initially used to handle and export the scans (Figure 3) as Digital Imaging and Communications in Medicine (DICOM) files.

### 2.4. Image Analysis

Image analysis was performed in three steps: segmentation, registration, and quantitative measurements.

CBCT scans were segmented by one observer using both manual and semiautomatic techniques and employing Amira Software (version 5.2.0, Visage Imaging Inc, Berlin/Germany). Image segmentation involved outlining mandibular contours and bone implants from the same subject at different time points. Surface meshes were extracted using the matching cubes algorithm [12], and Euclidian distances between the centroids of each implant were calculated. Segmented images of the mandibles at baseline and after ten weeks were rigidly registered by using bone fiducial implants as reference points. Furthermore, images were registered by applying affine followed by nonrigid techniques [13, 14], and the accuracy of the registration procedures was quantified by using the Hausdorff distance [15]. Surface meshes from the float images were extracted by using the algorithm proposed by Lorensen and Cline [16]. Floating images refer to the first image acquired in the beginning of the study from each subject. The resulting displacement fields generated from the registration procedure were applied to their respective surface meshes generated from the float images. Finally, the warped surface meshes were aligned by the Procrustes shape analysis method, allowing us to quantitatively assess the growth changes [17].

Displacement fields of the mandibles obtained at baseline and after 8 weeks were then computed and used to estimate a scalar field representing growth modification, with the baseline mesh as reference. Surface distances (mm) between two different time acquisitions within the same animal allowed quantification of the displacement fields describing mandibular growth. Scalar fields were then generated to describe growth, including magnitude and directional morphological changes. These indicated inward (blue) or outward (red) remodelling between overlaid structures, with negative or positive values, respectively. The negative or positive scalar fields indicated absence of displacement or minimal growth displacement. Visualisation through semitransparencies allowed an additional display of growth changes, clearly identifying the localisation, magnitude, and direction of mandibular displacements.

Image analyses were performed using MedInria Software Package (version 1.9.0, Asclepios, Inria Sophia Antipolis/France), VTK libraries (http://www.vtk.org/), and custom-made scripts developed in C++ and compiled with the GNU gcc 4.7.2 compiler. Mandibular volumes at baseline and after eight weeks were also calculated.

## 3. Results

All three rabbits completed the study. Mean body weight at the start of the study was 2.87 ± 0.03 kg; over the 65 days of the study, body weight steadily increased after a slight initial drop in weight due to surgery. However, the weight of the control subject was consistently higher than the two surgical animals throughout the study. The distances between the three mandibular implants changed slightly between baseline and after 10 weeks of growth (0.0 to 1.6 mm). The percentage change was greatest for the distance between the mandibular-symphysis and molar-region implants (range 0.8 to 1.6 mm, 10.6 to 11.7%). The three implants placed in the mandibular molar region showed little change (0 to 0.1 mm, 0 to 3.8%).

The mean error for image registration procedures was 0.37 mm and never exceeded 1 mm. An example of a scalar field representing the minimum Euclidian distances between two mandibular surface meshes is given in Figure 3. The sum of the Euclidian distances between the centroids of the three implants did not show any appreciable change (<0.20 mm) in one subject, whereas in another subject one out of the three implants was displaced by 1.60 mm.

From measurements made in one control and two experimental animals, vertical growth was comparatively more prominent than sagittal and transverse growth in the rabbit mandible. Implant-based overlay of mandibles at baseline and after 10 weeks suggests that the majority of the remodelling process occurred at the mandibular ramus with bone apposition posteriorly and vertical growth at the mandibular condyle. No bone resorption could be observed at the anterior aspect of the ramus. The anterior aspect of the condyle, however, appeared to be an area of bone resorption during the study period. The mandibular molar region was shown to be the most stable area for superimposition in the rabbit model.

**Figure 4:**
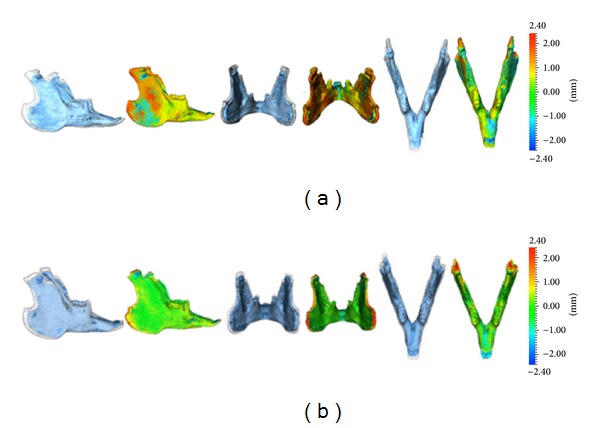
Comparison between Implant-based registration (a) and registration followed by Procrustes analysis (b) in one test animal. Surface meshes of the mandible obtained at baseline (blue) and after eight weeks (grey) were made transparent and then superimposed. Scalar fields were used to assess morphological changes using a color-coded quantitative representation (color map): Red indicating bone outward/deposition, green indicating small or no changes and blue indicating inwards/resorption. Note the greater amount of remodelling changes occurring at the mandibular ramus revealed by the implant method as compared to the registration/Procrustes method.

**Figure 5:**
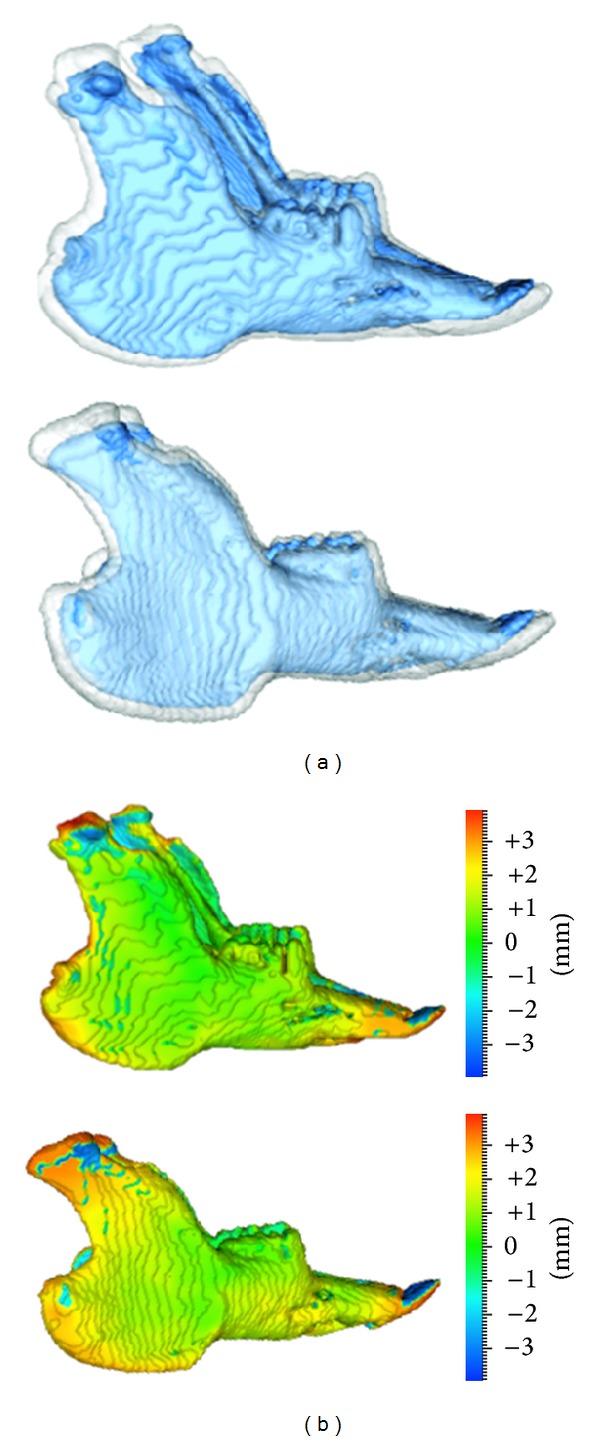
Surface meshes of mandibles obtained at baseline (blue) and after eight weeks (grey) in the other two rabbits. Superimpositions were obtained by registration of meshes followed by Procrustes analysis. Note the consistent patterns of morphological changes as revealed by color-coded maps, in the two mandibles.

Following registration, the transparent rabbit mandible overlays were analysed by the Procrustes method, producing colour-coded scalar fields (Figures 4(b), 5(a), and 5(b)). The resulting scalar fields allowed 3D assessment of the vertical, sagittal, and transverse growth remodelling changes. During the study period, the overall volumes of the three rabbit mandibles increased by 3.0 cm^3^ (36.9%), 3.4 cm^3^ (45.8%), and 3.7 cm^3^ (46.3%), respectively.

## 4. Discussion

Reliable methods for assessing mandibular growth in 3D are much needed, especially for evaluation of growth modification treatments. We have tested an animal method for investigating mandibular growth in 3D. This is based on Björk's original 2D radiographic implant method [3] and uses bone implants as fiducial markers and CBCT as the imaging modality. In comparison with Björk's study which used 1.5 mm by 0.5 mm long pins as markers, our implants were spherical and 1 mm in diameter, allowing multidirectional superimposition.

This method was successfully used to assess 3D remodelling patterns of a rabbit mandible. For one of the three rabbits, the implant in one site was significantly displaced during growth, so that an accurate overlay of the images based on implants was not possible. Implant failure was most likely due to interference with the eruption path of mandibular teeth, which occupy most of the mandibular bone. We suggest that more than three markers need to be placed, in order to compensate for accidental displacement of bony markers during growth.

To the best of our knowledge, this is the first investigation describing 3D mandibular growth during normal skeletal maturation in rabbits using CBCT-imaging. Therefore direct comparison with other rabbit findings could not be performed. The remodelling pattern observed in our study was consistent with the existing knowledge of mandibular growth in rabbits [18], encompassing condylar growth, deposition of bone on the labial and lingual sides of the body of the mandible, and growth of the ramus in vertical, posterior/causal, and superior directions. Compared to the rest of the mandible, the area below the lower molars was minimally affected by the remodelling process and therefore most suited to the placement of the fiduciary markers. This is consistent with histological findings of a “neutral” region described in previous research [19]. Unlike the human mandible [3], the remodelling of the rabbit mandibular ramus did not involve resorption of the anterior border, at least during the observation period of this study.

In our study, 3D overlays and corresponding growth remodelling maps were obtained using two different registration methods; the first method was based on the use of bone fiducial markers (implants), whereas the second was based on image registration of the entire volumetric data set of the segmented mandible followed by Procrustes superimposition. Procrustes analysis is a widely used method in geometric morphometrics and involves alignment of models by optimal translation, rotation, and scaling of objects [20]. Whilst the latter registration method was accurate and could be successfully used to assess gross morphological changes occurring during growth, the biological significance of this superimposition remains dubious. Using the implant-based method, we identified regions that showed less variation during growth; the next step of our research will be that of assigning a higher weighing to these regions during Procrustes superimposition. In this way, an automatic registration procedure could produce 3D superimpositions that are biologically more valid. The development of a biologically weighted registration procedure is the subject of our ongoing research.

This pilot study reports our initial analysis of 3D mandibular growth in a rabbit model. Although the use of a small sample size may have limited our ability to produce normative data sets and make generalisation from findings, the study does support feasibility of the model. Within the limitations of the present research, we found that this model permitted the three-dimensional assessment of mandibular growth based on stable fiducial markers. The proposed method is feasible and represents a promising approach for future experimental treatment intervention studies. This will allow a three-dimensional appreciation of normal mandibular and craniofacial growth and may provide new insights into current knowledge of craniofacial growth and the effects of orthodontics and dentofacial orthopaedic appliances.
